# Global reconstruction of historical ocean heat storage and transport

**DOI:** 10.1073/pnas.1808838115

**Published:** 2019-01-07

**Authors:** Laure Zanna, Samar Khatiwala, Jonathan M. Gregory, Jonathan Ison, Patrick Heimbach

**Affiliations:** ^a^Department of Physics, University of Oxford, Oxford OX1 3PU, United Kingdom;; ^b^Department of Earth Sciences, University of Oxford, Oxford OX1 3AN, United Kingdom;; ^c^Met Office Hadley Centre, Exeter EX1 3PB, United Kingdom;; ^d^National Centre for Atmospheric Science–Climate, University of Reading, Reading RG6 6BB, United Kingdom;; ^e^Institute for Computational Engineering and Sciences, The University of Texas at Austin, Austin, TX 78712;; ^f^Jackson School of Geosciences, The University of Texas at Austin, Austin, TX 78712

**Keywords:** ocean heat content, Earth’s energy imbalance, sea-level rise, climate change, ocean processes

## Abstract

Most of the excess energy stored in the climate system due to anthropogenic greenhouse gas emissions has been taken up by the oceans, leading to thermal expansion and sea-level rise. The oceans thus have an important role in the Earth’s energy imbalance. Observational constraints on future anthropogenic warming critically depend on accurate estimates of past ocean heat content (OHC) change. We present a reconstruction of OHC since 1871, with global coverage of the full ocean depth. Our estimates combine timeseries of observed sea surface temperatures with much longer historical coverage than those in the ocean interior together with a representation (a Green’s function) of time-independent ocean transport processes. For 1955–2017, our estimates are comparable with direct estimates made by infilling the available 3D time-dependent ocean temperature observations. We find that the global ocean absorbed heat during this period at a rate of 0.30 ± 0.06 W/$m^{2}$ in the upper 2,000 m and 0.028 ± 0.026 W/$m^{2}$ below 2,000 m, with large decadal fluctuations. The total OHC change since 1871 is estimated at 436 ± 91 $\times 10^{21}$ J, with an increase during 1921–1946 (145 ± 62 $\times 10^{21}$ J) that is as large as during 1990–2015. By comparing with direct estimates, we also infer that, during 1955–2017, up to one-half of the Atlantic Ocean warming and thermosteric sea-level rise at low latitudes to midlatitudes emerged due to heat convergence from changes in ocean transport.

## Significance

Since the 19th century, rising greenhouse gas concentrations have caused the ocean to absorb most of the Earth’s excess heat and warm up. Before the 1990s, most ocean temperature measurements were above 700 m and therefore, insufficient for an accurate global estimate of ocean warming. We present a method to reconstruct ocean temperature changes with global, full-depth ocean coverage, revealing warming of 436 $\times 10^{21}$ J since 1871. Our reconstruction, which agrees with other estimates for the well-observed period, demonstrates that the ocean absorbed as much heat during 1921–1946 as during 1990–2015. Since the 1950s, up to one-half of excess heat in the Atlantic Ocean at midlatitudes has come from other regions via circulation-related changes in heat transport.

**Fig. 1.**
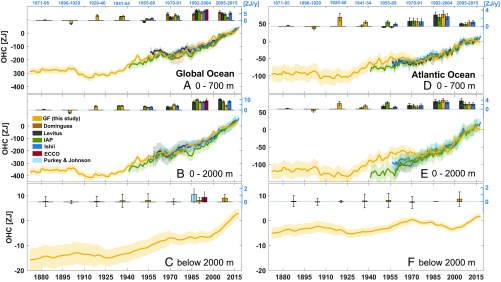
Global and Atlantic OHC timeseries and trends for GF and observational estimates relative to 2006–2015. Timeseries of global (*A–C*) and Atlantic (*D–E*) OHC changes in zetajoules (1 ZJ = 10^21^ J): (*A* and *D*) top 700 m, (*B* and *E*) top 2,000 m, and (*C* and *F*) below 2,000 m. The OHC timeseries include the reconstruction based on GFs (orange) and direct measurements from the NCEI (2) (black), the IAP (1) (green), Ishii et al. (20) (blue), and Domingues et al. (updated from refs. 21 and 22) (brown). The latitudinal range for all products used here is 80° S to 80° N, except for the product from Domingues et al. (21), which uses 65° S to 65° N. The shading represents the uncertainty associated with each estimate (*Materials and Methods*). *Insets* above each panel represent the linear trends and associated error (zetajoules per year) over different periods for each best estimate available (see text). For the global ocean (*A–C*), we include trends from the ECCO-GODAE solution (red) and for the deep ocean (*C*) the updated estimates from refs. 1, 23, and 24 (cyan).

The ocean, due to its large heat capacity, has absorbed more than 90% of the heat gained by the planet between 1971 and 2010, with around 290 $\text{ZJ}\left(1\text{ZJ}=10^{21}\text{J}\right)$ contained in the top 2,000 m (Fig. 1) (1, 2). However, near-global data coverage has been only achieved since 2006 with the full deployment of Argo profiling floats in the upper 2,000 m (3). Earlier observations were geographically more sparse, restricted to shallower depths (except for shipborne conductivity, temperature, and depth casts), and insufficient to permit an accurate global estimate of ocean heat content (OHC) before the 1950s (an extensive discussion of OHC estimates and associated errors is in ref. 1). Global OHC, global mean sea surface temperature (SST), and sea level have been incontestably rising in the past several decades (4, 5). However, there are significant regional variations in their patterns owing in part to the differing imprint of the forcing on the ocean surface and heat redistribution by ocean processes, with the latter being particularly difficult to measure or infer (6).

To complement and compare with the direct estimates of OHC change made by infilling the observational datasets of interior temperatures, we have derived an independent global and regional reconstruction of annual mean OHC. Our method combines time-dependent SSTs with a time-independent representation of ocean transport processes. Our use of SST records enables us to estimate OHC for the full depth going back to 1871. The comparison of our OHC reconstruction with direct estimates enables us to also infer the role of changes in ocean transport in shaping patterns of OHC, in particular in the Atlantic Ocean where observations are the densest and where the contrast in storage between low and high latitudes is large (see Fig. 3).

## Historical Passive OHC Reconstruction

Using observation-based products, we quantify spatiotemporal variations in passive temperature and OHC change (7, 8) from 1871 to 2017. By “passive,” we mean that we assume temperature anomalies to behave as a passive tracer (7) set at the sea surface and carried into the ocean interior via time–mean climatological ocean transport processes, which comprise advection, mixing, and turbulent diffusion (9). These time–mean processes are represented via Green’s functions (GFs) (10, 11), which connect surface properties to those in the ocean interior. The SST anomaly, when propagated into the ocean interior by the GFs, is assumed not to affect the transport processes; that is, the GF is constant except for variations associated with the seasonal cycle (*Materials and Methods* and *SI Appendix*). Interannual variability in the estimated OHC will, therefore, only be inherited from SST variability under the assumption that the ocean circulation was in equilibrium in 1871 (*Materials and Methods*). Note that the imposed SST variability is not solely due to atmospheric forcing but also, due to a varying ocean circulation. Hence, the OHC fields produced here include limited influence of changing ocean transport despite the assumption of a time–mean GF.

The time–mean GFs are derived from the Estimating the Circulation and Climate of the Ocean (ECCO)-Global Ocean Data Assimilation Experiment (GODAE) (12) ocean state estimate (1992–2004), combining an ocean model and observations, while the observed annual mean SST anomalies are taken from HadISST v1 (13). To reduce potential local biases and computational costs, we average the SST anomalies over 26 large spatial patches (*SI Appendix*, Fig. S1) (14) selected according to their climatological density in each ocean basin, and we compute the GFs over zonally averaged 10°-latitudinal bands and 22 vertical levels. The patterns and magnitudes of the ECCO-GODAE time–mean barotropic, Sverdrup, and overturning transport are in agreement to those derived solely from observations, including using different time periods (15–17). However, biases associated with shallow to deep pathways are likely present (18, 19). To partially account for uncertainties associated with both the imperfect time–mean ocean transport from the ECCO-GODAE and the incomplete SST coverage, especially as we go farther back in time, we perturb our GFs and use different realizations of SST timeseries. The GFs are perturbed by 20% in the upper 2,000 m and 40% below 2,000 m to mimic the uncertainty in ocean transport from the ECCO-GODAE (18, 19) and from observationally based products (6), and we convolve these GFs with 10 different realizations from HadISST v2.0 (*SI Appendix*).

The timeseries and trends of global OHC reservoir reconstructed from time–mean GFs and time-dependent SSTs (Eq. **1**) are compared with a suite of observational estimates (Fig. 1 *A*–*C*). The OHC observational datasets (detailed in *Materials and Methods*) (1, 2, 20–24) are those routinely analyzed in intercomparison studies (25–27), which combine ocean interior measurements together with different methods for mapping and infilling of temperature (*SI Appendix*). The uncertainty associated with each observational dataset has been well documented (1, 27), with mapping identified as an important source of error in estimates of OHC. We, therefore, use a range of observational products to span the range of uncertainty. All warming rates are expressed in watts per square meter over the Earth’s surface area (5.1 $\times 10^{14}\;m^{2}$).

The rate of warming of the global ocean, calculated from a linear least squares fit, using the GF reconstruction between 1955 and 2017 is estimated at 0.22 ± 0.05 and 0.30 ± 0.06 W/$m^{2}$ in the upper 700 and 2,000 m, respectively. For all time periods selected, the GFs estimates are situated within the range of the individual observational estimates and those derived from reanalyses products (28, 29). The ensemble means, derived from observational products, and associated errors using one SD are 0.21 ± 0.03 W/$m^{2}$ (top 700 m) and 0.30 ± 0.01 W/$m^{2}$ (top 2,000 m). The uncertainty derived for the trends is likely optimistic (17), especially given the uncertainty associated with the sparsity of data in the earlier part of the record. Below 2,000 m, the rate of warming is comparable with recent repeat hydrography estimates (23, 30, 31) and is on the order of 0.028 ± 0.026 W/$m^{2}$ (Fig. 1*C*), with a rate of 0.06 ± 0.04 W/$m^{2}$ since the early 2000s. Unlike in observations, the reconstructed warming below 2,000 m is not statistically significant during the period 1992–2004. This discrepancy between the reconstruction and direct measurements could be because of the poor observational sampling, a poor representation of shallow to deep pathways in the ECCO-GODAE, or ocean transport changes not captured by a steady GF with time-varying SSTs.

The global full-depth OHC from 1871 to present is estimated at 436 ± 91 ZJ. The reconstructed OHC increase during 1921–1946 (145 ± 62 ZJ) is comparable with change estimated during 1990–2015 (153 ± 44 ZJ). Rates of warming are significant in the upper 2,000 m, despite the large decadal fluctuations. However, the deep ocean warming signal below 2,000 m has only emerged in the recent decades. Between 1960 and 2017, the increase in ocean storage of 323 (±66) ZJ led to a rate of global mean thermosteric sea-level change (*Materials and Methods*) of 0.9 ± 0.1 mm/y compared with 0.8 ± 0.3 mm/y based on the ensemble mean of the direct observational estimates used in our study. For 1990–2017, the rate from the GF reconstruction was 1.2 ± 0.2 mm/y compared with 1.1 ± 0.3 mm/y from direct estimates. For context, thermal expansion of the ocean contributed 40% of global mean sea-level rise over 1971–2010 (25, 28, 32–34).

The GF OHC estimates are also in close agreement with direct estimates in individual basins, with those in the well-sampled Atlantic always being within the observational uncertainties between 1971 and 2017 and showing the smallest relative errors (Fig. 1 *D*–*F*). The largest discrepancies between the GF and direct OHC estimates are in the Indian Ocean, where the circulation estimated by the ECCO-GODAE is known to have the largest errors and where observations are sparser (*SI Appendix*, Fig. S2) (figure 10 in ref. 16). In the upper 2,000 m of the Atlantic Ocean, the linear trends from the GFs estimates are 0.14 ± 0.05 W/$m^{2}$ over the period 2005–2015, 0.24 ± 0.05 W/$m^{2}$ over 1992–2004, and 0.06 ± 0.04 W/$m^{2}$ over 1970–1991 (Fig. 1*E*). The observational estimates for the same periods are 0.17 ± 0.05, 0.20 ± 0.01, and 0.10 ± 0.01 W/$m^{2}$, respectively. Over these different periods, the GF and observational estimates are within a few percent of each other. Before 1970, the uncertainty among the observational datasets is large. A similar behavior as for the upper 2,000 m is observed for the upper 700 m (Fig. 1*D*). The passive OHC increase in the Atlantic since 1971 is dominated by the upper 700 m (51.2 ± 4.9 ZJ), with a growing storage between 700 and 2,000 m between 2000 and 2015 (17.9 ± 5.4 ZJ). The increase in OHC below 2,000 m is relatively small (35, 36), about 3.4 ZJ since the early 1990s, but fairly uncertain (±3.2 ZJ) with variations up to 5 ZJ over several decades.

Our method provides a complementary estimate of basin-integrated OHC and thermosteric sea-level change. The agreement of our results with observations suggests that most of the basin-integrated heat storage in the Atlantic is passive, meaning that it is explained almost entirely by the propagation of SST anomalies via the time–mean transport into the ocean interior and consistent with our use of a constant GF. We estimate that only about up to 5% of the Atlantic OHC change over the last 45 y may be due to unaccounted changing ocean processes or errors in methods. Changes in ocean processes, possibly induced by wind or buoyancy forcing, can include an exchange with the (poorly sampled) deep ocean or with other basins, such as the Arctic and Pacific Oceans. However, the discrepancies may be due to inaccuracies in the ECCO-GODAE solution, inaccuracies in the GF estimates, or observational errors (*Materials and Methods* and *SI Appendix*).

## Cumulative Ocean Uptake

The passive temperature anomaly computed by our approach can be quantitatively partitioned according to where at the surface the heat was taken up (Eq. **2**). This is a consequence of GFs being interpreted as the fraction of water at any interior location that was last in contact with a given surface region (10).

**Fig. 2.**
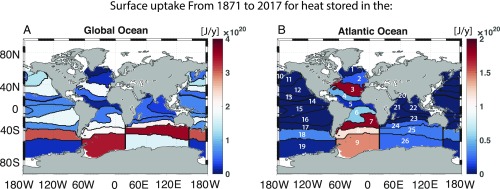
Cumulative heat uptake from 1871 to 2017 (joules per year) shown for each patch (numbered here and shown in *SI Appendix*, Fig. S1), contributing to the integrated passive heat storage (*A*) globally and (*B*) in the Atlantic Ocean. Note the different scales for the two panels.

A considerable portion of the passive OHC stored in the global oceans (Fig. 2*A*) and the Atlantic Ocean (Fig. 2*B*) since 1871 was taken up in midlatitudes and in the Southern Ocean (37). For the global storage of passive heat, 72 $\times 10^{19}$ J/y was absorbed in the southern Atlantic Ocean (patches 7–9, with patch 9 enclosing the Weddell Sea), 55 $\times 10^{19}$ J/y was absorbed in the southern Pacific Ocean [patches 16–19, where patch 19 (which encloses the Ross Sea) contributes only 2 $\times 10^{19}$ J/y], and 73 $\times 10^{19}$ J/y was absorbed in the southern Indian Ocean (patches 24–26). Smaller but yet substantial amounts of passive heat can be traced back to the midlatitude North Atlantic (patch 3) and North Pacific (patches 11 and 12), with cumulative uptake rates of 18 $\times 10^{19}$ and 30 $\times 10^{19}$ J/y, respectively. For heat stored in the Atlantic Ocean (Fig. 2*B*), the uptake occurred mostly south of 30° S in the Atlantic (patches 7–9), accounting for roughly 45 $\times 10^{19}$ J/y, and in the midlatitude North Atlantic (patch 3), with another 18 $\times 10^{19}$ J/y. The subtropics (patches 4 and 6) contribute about 10 $\times 10^{19}$ J/y. We estimate that an additional 6 and 3 $\times 10^{19}$ J/y have originated from the southern Indian (patches 24–26) and the southern Pacific Oceans (patch 18), respectively. Therefore, all of the passive heat taken up in the midlatitudes of the Pacific and Atlantic Oceans remains in the respective ocean basins. About 63% of the heat absorbed in the South Atlantic is found in the Atlantic basin.

**Fig. 3.**
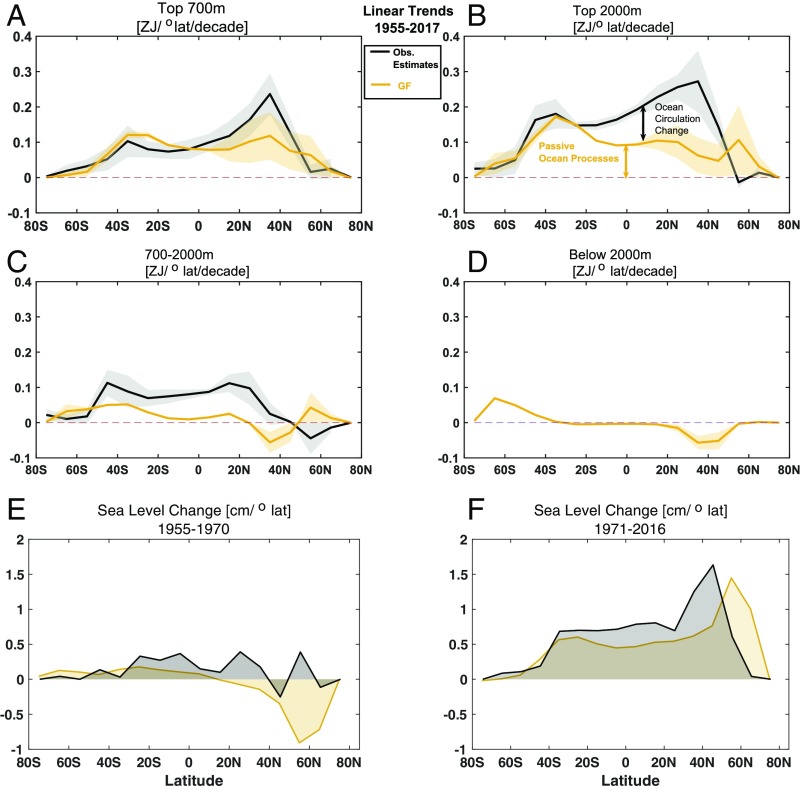
OHC and sea-level trends in the Atlantic Ocean as a function of latitude. Atlantic OHC linear trends calculated over 1955–2017 (ZJ per degree latitude per decade) as a function of latitude for GF (orange) and observational estimates (black) and for different depth ranges: (*A*) top 700 m, (*B*) top 2,000 m, (*C*) 700–2,000 m, and (*D*) below 2,000 m. The average uncertainty (shading) is calculated using the signal to noise ratio from the different datasets, thereby partially including both the departure of the signal from the linear trend over a decade and the uncertainty in the trends from the different observational products. *E* and *F* show the difference in sea level (centimeters per degree latitude) estimated using the upper 2,000-m OHC during the periods 1955–1970 and 1971–2016, respectively. The difference is estimated using an average of the first and last 5 y in each period.

High latitudes, where deep water formation occurs, are negligible contributors to the net passive uptake over 1871–2017 due to the large decadal variability signal in SST (*SI Appendix*, Fig. S1), leading to a lack of discernible trends in OHC in these regions (Fig. 3 and *SI Appendix*, Fig. S3). While heat originating at high latitudes can penetrate to great depths, the majority of the passive uptake is not through this pathway but through midlatitude dynamics and thermodynamics. For passive heat uptake estimates under a time–mean GF assumption, changes in ocean processes are not fully captured in these areas (*SI Appendix*, Fig. S3).

### Distribution of Ocean Heat Storage and Transport

When averaged over the World Ocean, changes in ocean processes have no effect on global OHC (via conservation of heat). Here, we find that changes in ocean transport since 1871 have a negligible effect on the basin integrated Atlantic OHC as well. However, this is not the case when considering latitudinal patterns of heat storage. Any differences in the spatial pattern of heat storage between the GF reconstruction and observational products indicate a change in ocean processes leading to heat convergence or divergence (i.e., that the GF assumption of constant transport processes is not accurate). For example, we expect that changes in temperature will alter buoyancy gradients and therefore, the large-scale ocean circulation.

To probe the role of ocean circulation changes in shaping patterns of heat storage in the Atlantic, we examine the linear trends in all observational datasets and in the GF estimates from 1955 as a function of latitude for different depth ranges (Fig. 3). The passive OHCs from GF estimates (Fig. 3 *A* and *B*, orange curve) and direct observations (Fig. 3 *A* and *B*, black) have similar latitudinal profiles in the Southern Ocean between 80° S and 20° S, with a peak of 0.18 ZJ per degree latitude per decade around 40° S in the upper 2,000 m. North of 20° S, the signature of a changing ocean circulation is more prominent.

Between 20° S and 50° N, the trends in passive OHC are consistently weaker than in direct estimates over the upper 2,000 m, with difference ranging from 0.05 ZJ per degree latitude per decade at 20° S to 0.1 ZJ per degree latitude per decade at 30° N (Fig. 3*B*). Changes are also apparent north of 50° N, but these are not statistically significant due to the large uncertainty in the observational products and the presence of strong variability, especially in the upper 700 m (*SI Appendix*, Fig. S3). However, the peak OHC change in the upper 2,000 m of the North Atlantic based on GF estimates occurs in midlatitudes, with a maxima of 0.1 ZJ per degree latitude per decade around 45° N to 60° N (Fig. 3*B*, orange curve) compared with 0.3 ZJ per degree latitude per decade around 30° N to 40° N in observations (Fig. 3*B*, black). Most of the signature of ocean circulation changes is found at intermediate depths between 700 and 2,000 m (Fig. 3*C*), which have higher signal to noise ratios than surface properties. The observed trends are of opposite signs to those of the GF-based estimates poleward of 20° N. Below 2,000 m, in the North Atlantic, passive heat storage is negative and decreases at a rate of 0.08 ZJ per degree latitude per decade north of 20° N. This result is consistent with ref. 38 over the 1988–2007 period but not with observed warming during 1957–1987. South of 40° S, there is a trend in deep heat storage of 0.09 ZJ per degree latitude per decade since 1955 (Fig. 3). Our analysis suggests that changes in ocean processes may have accelerated the warming rate in the top 2,000 m in low latitudes and midlatitudes of the Atlantic basin by shifting meridionally the pattern of ocean warming.

## Implications and Discussion

Based on the comparison between the GF and direct estimates, we can assess how changes in ocean transport have influenced patterns of ocean heat storage. Our results suggest that, if the heat storage was entirely determined by the climatological transports, its magnitude over the last 40 y would be less pronounced in low latitudes and more pronounced north of the Gulf Stream than observed, despite the large uncertainty in all estimates in these regions. We attribute 1.3 ZJ per degree latitude per decade of the heat storage between 20° N and 50° N in the North Atlantic to redistribution by changes in ocean transport. We infer that, during 1955–2017, one-third to one-half of the warming in the Atlantic basin between 20° S and 50° N was due to heat convergence from ocean circulation changes.

The changes in ocean transport inferred here also affect regional thermosteric sea-level change. During 1971–2017, changes in transport contributed to one-half of the observed 1.8-cm sea-level rise at 30° N to 40° N in the Atlantic (due to 0.3 ZJ per degree latitude per decade of heat convergence from ocean circulation changes) but led to a reduction poleward of 50° N (Fig. 3*F*). Changes in ocean transport are responsible for up to 1 cm of thermosteric sea-level change in the North Atlantic (Fig. 3 *E* and *F*). This result is consistent with the magnitude of changes inferred from other observational studies (39).

Several hypotheses, which are not mutually exclusive, could account for such OHC patterns arising from circulation changes. Changes in midlatitudes wind forcing (40, 41) can excite an upper ocean adjustment with a signature in both gyres, modifying the horizontal exchange of heat between the subtropical and subpolar regions. This signature can potentially be amplified by deep climatological mixed layers at high latitudes. The inferred latitudinal changes in heat storage could also be induced by changes in the upper branch of the meridional overturning circulation (MOC)—including both horizontal and vertical transport—driven by wind and buoyancy forcing and mixing (9). This hypothesis is consistent with a reduction in North Atlantic deep water formation and slowing of the MOC, leading to an increase in OHC at low latitudes (42–44). Lastly, different forcings at the surface or inflows of water from other basins below 2,000 m at low and high latitudes can act together to change heat storage in these regions independently from one another.

Our results highlight that the substantial amounts of heat accumulated in the ocean and associated sea-level rise can be influenced by ocean circulation changes and low- to midlatitude air–sea interactions. Changes in wind and air–sea fluxes, including those due to cloud feedbacks, may play an increasing role under anthropogenic climate change as suggested by the shape of passive OHC trends in the Atlantic during 1871–1955 versus 1955–2017 (Fig. 3 and *SI Appendix*, Fig. S4). Future changes in ocean transport could have severe consequences for regional sea-level rise and the risk of coastal flooding. Monitoring and understanding OHC change and the role of circulation in shaping the patterns of warming remain key to predicting global and regional climate change and sea-level rise.

## Materials and Methods

### Observation-Based Datasets.

We use the ECCO-GODAE (ECCO version 2) solution (12) to derive the time–mean transport matrix and GFs (*GF Reconstruction: OHC and Thermosteric Sea Level*). The ECCO-GODAE solution combines a state-of-the-art ocean general circulation model, the MITgcm (45), at a 1° horizontal resolution and an adjoint-based assimilation with all available global ocean datasets over the period ranging from 1992 to 2004. The domain extends from 80 °S to 80 °N. To compare our GF-based OHC estimates with direct observations, we use the following data products. From the National Centers for Environmental Information (NCEI), we use the global analyzed gridded fields of OHC from 1955 to 2017. We use yearly averaged fields for the depth range 0–700 m and pentadal averages for 0–2,000 m (2, 46). From the Institute of Atmospheric Physics (IAP) and the objective analysis of Ishii et al. (20), we use the yearly average ocean temperatures from the 41 vertical levels down to 2,000 m between 1940 and 2016 (1) and between 1955 and 2017, respectively. All datasets are gridded products with 1° latitude–longitude horizontal resolution. For the upper 700 m, we also used the global updated OHC estimates from Domingues et al. (21, 22). The SST datasets used as surface boundary conditions (*SI Appendix*, Fig. S1) are from The Met Office Hadley Center’s Sea Ice and Sea Surface Temperature Dataset HadISST, which combines fields of SST on a 1° latitude–longitude grid from 1871 to 2017. We use both HadISST v1 (13) and v2.0 (47); the latter offers a 10-member ensemble, which is used as part of the uncertainty quantification of our OHC estimates.

### GF Reconstruction: OHC and Thermosteric Sea Level.

We calculate the GFs over zonally averaged 10° latitudinal bands and 22 vertical levels from the ECCO-GODAE state estimate using the Transport Matrix Method (48, 49). The transport matrix and associated GFs represent tracer transport, including resolved and parametrized advection, and parametrized subgrid-scale mixing processes. The GFs are a representation of ocean pathways in terms of a distribution of timescales and therefore, probability. The GFs reflect the steady-state linearized ocean transport and can be used to propagate any tracer from the surface into the interior, assuming that the tracer does not affect ocean transport. The 26 surface patches used to connect the surface to the interior are defined based on their climatological density in each basin (14), and they are shown in *SI Appendix*, Fig. S1. The temporal evolution of area-weighted SST anomalies relative to 1871 in each of the 26 patches is also shown in *SI Appendix*, Fig. S1. Positive trends in SSTs, especially since 1950, are apparent in most regions with a few exceptions: mostly patches 1 and 2 in the North Atlantic and patches 12 and 16 in the Pacific, which only exhibit low-frequency fluctuations. The use of large-scale averages for SST patches and GFs, in addition to reducing computational cost, substantially minimizes the possible impact of model-dependent transport on the OHC. We estimate the OHC in the interior over a volume $V_{R}$ relative to 1871 using the following convolution:

$$OHC\left(V_{R},t\right)=\underset{V_{R}}{\int }dr\;c_{p}\;\rho \left(r,t\right)\overset{t}{\underset{1871}{\int }}dt\prime \underset{S}{\int }dr\prime G\left(r,t-t\prime ;r\prime \right)\;T^{S}\left(r\prime ,t\prime \right),$$ [1]

where $c_{p}=3,992\;J\;{kg}^{-1}C^{-1}$ is the specific heat capacity, $T^{S}$ is the SST anomaly at location r′ and time t′ relative to 1871, and S is the surface of the ocean. The operator G(r,t−t′;r′) is defined as the GF (10, 11) that propagates SST anomalies $T^{S}$ at a location r′ and time t′ to the interior at location r and time t. The GF has no time dependence, and the convolution (Eq. **1**) assumes that the SST anomalies before 1871 must be zero; therefore, the ocean circulation must be in equilibrium. The density ρ(r,t) in kilograms per cubic meter is calculated using temperature T and salinity S at (r,t) from the Ishii dataset (20) and the Gibbs Seawater Oceanographic Toolbox (50). The surface integral in Eq. **1** is discretized over the 26 surface regions, each denoted by $r_{i}^{\prime }$ with i=1,…,26. The integrated cumulative heat uptake (Fig. 2) through a patch i from 1871 to time t is such that

$$OHU\left(r_{i}^{\prime },V_{R},t\right)=\underset{V_{R}}{\int }\;dr\overset{t}{\underset{1871}{\int }}dt\prime \;c_{p}\;\rho \left(r,t\right)\;G\left(r,t-t\prime ;r_{i}^{\prime }\right)\;T^{S}\left(r_{i}^{\prime },t\prime \right),$$ [2]

where $r_{i}^{\prime }$ is the discretized surface patch i with surface area $A_{i}$. Consider that $OHC=\rho c_{p}\;V\Delta T$, with ΔT being the change in ocean temperature in a region with a volume V. For a change in temperature, the associated induced change in volume is ΔV=VαΔT. Therefore, $\Delta V=A\;\Delta SSH=\frac{\alpha OHC}{\rho c_{p}}$, where ΔSSH is the thermosteric sea-level change, A is the surface area, and α=−1/ρ ∂ρ/∂T is the thermal expansion coefficients calculated using Gibbs Seawater Oceanographic Toolbox (50) and Ishii temperature and salinity analyses (20). The thermosteric sea-level change can be expressed as $\Delta SSH=\frac{\alpha OHC}{A\rho C_{p}}$. The calculations for OHC and ΔSSH using a reference density or pentadal salinity and temperature anomalies from the NCEI (46) resulted in changes of less than 2% in our estimates of integrated OHC and thermosteric sea-level change.

## Supplementary Material

Supplementary File
