# Supplementary material for: Global reconstruction of historical ocean heat storage and transport
Source: Proc Natl Acad Sci U S A. 2019 Jan 7;116(4):1126–31. doi: 10.1073/pnas.1808838115 (PMC6347704; doi:10.1073/pnas.1808838115)
Supplement: Supplementary File [file pnas.1808838115.sapp.pdf]

Supporting Information (SI Appendix): Global reconstruction of historical ocean heat storage and transport

Zanna et al. 10.1073/pnas.1808838115

Here we present further information on the availability of the products used in our study, the uncertainty quantification in GF and datasets, and the regional time-dependent OHC estimates.

A. Data Availability. In addition to the references provided in the main text and the *Materials and Methods* section, we are listing the links to all datasets used in the present study. The ECCO-GODAE data can be downloaded from http://www.ecco-group.org/products.htm. The Hadley centers SST datasets are at https://www.metoffice.gov.uk/hadobs/hadisst/. The NCEI OHC and salinity data are available at https://www.nodc.noaa.gov/OC5/3M_HEAT_CONTENT/heat_global.html and https://www.nodc.noaa.gov/cgi-bin/OC5/SAL_ANOM/showfiganom.pl?action=start, respectively. The IAP, Ishii and Domingues data are available from http://159.226.119.60/cheng/, https://climate.mri-jma.go.jp/pub/ocean/ts/v7.2/doc/00README, and http://www.cmar.csiro.au/sealevel/thermal_expansion_ocean_heat_timeseries.html, respectively.

B. Code Availability. The TMM code and climatological transport matrices extracted from the ECCO-GODAE state estimate are available on GitHub https://github.com/samarkhatiwala/tmm, and the GFs are available from the corresponding author upon request.

C. Error Estimates: GFs reconstructions. The OHC reconstruction from GFs presented in this study is subject to two primary sources of uncertainty: 1) errors in the imperfect representation of ocean transport processes (e.g., advection, mixing) in ECCO-GODAE, which can be due to the model resolution and parametrizations and/or the lack of data in some regions - this will then translate into errors in the computed GFs and pathways between the ocean interior and the surface; and 2) errors in SSTs due to poor spatial and temporal sampling, particularly outside the Atlantic basin and in the early part of the record. In this study, we have devised a strategy, described below, to both minimize the dependence of the OHC estimates on model and observational biases, and also to partially account for the uncertainty associated with the imperfect knowledge of ocean transport and SSTs from data and ECCO-GODAE.

We select broad areas both at the surface for SSTs and in the ocean interior for the GFs, which led to more robust patterns of OHC and associated uncertainties despite reducing the horizontal resolution of the estimates. The transport matrix and GFs, themselves, are subject to uncertainty associated with the ECCO-GODAE representation of ventilation pathways, which are affected in part by model resolution and numerical mixing. The patterns and magnitude of the ECCO-GODAE time-mean barotropic and Sverdrup transport are in agreement to those derived directly from observational products (1–4). However, a comparison of simulated bomb radiocarbon with observations suggests that shallow–to–deep exchange in ECCO-GODAE may be too efficient (5, 6). Despite this bias, the inventory and spatial distribution of anthropogenic CO_2 simulated by ECCO-GODAE have been shown to be in line with observational estimates (6, 7). In addition, a detailed analysis (8) using a more recent version of ECCO, which is not qualitatively different from previous ECCO versions (except for the longer period of assimilation), produces abyssal heat content changes at high Southern latitudes that are consistent with those of (9) (as also shown here in Fig. 1C).

Nonetheless, ECCO-GODAE pathways are derived from an ocean model at 1° horizontal resolution which inevitably possesses some biases, despite being constrained by observations. To include this uncertainty, without having to recalculate the GFs for several ocean reanalyses, which is computationally challenging, we have opted to perturb our estimates of the GFs. The uncertainty in observationally-based, basin-averaged GFs has been estimated to be O(10-20%) (10). In addition, crude estimates derived from previous studies (5, 6) suggest O(20-30%) error in shallow to deep exchange of water. Finally, comparison of ocean reanalysis products (11) shows a 20 to 30% spread in the amplitude of the upper and lower overturning cells. Therefore, we perturb the GFs by 20% in the upper 2000 m and 40% below 2000m in an attempt to represent the transport uncertainty derived from ocean reanalyses, and tracer-based observational estimates. The perturbations are applied while imposing mass conservation by renormalizing the GFs. This uncertainty representation is potentially conservative and will be investigated in future work by using GFs estimated from different observation-based products (e.g., other ECCO state estimates, or direct climatological products such as GLODAP), and/or over different time periods.

Finally, we convolve the GFs with 10 different realizations from HadISST v2.0, rather than using HadISST v1 alone to include uncertainty in surface boundary conditions. The ensemble-mean estimate of OHC, from 1955 onwards, based on HadISST v2.0 is only within 2% of the one based on HadISST v1. The error prior to 1955 is large due to the reduced availability of surface temperatures. Using two additional SST estimates from the NOAA Extended Reconstruction SSTs V4 (12) or from COBE (13) did not result in different OHC estimates (less than a few percents change) and those estimates are therefore left out of the present study.

Overall, the OHC and associated errors from the GFs are comparable to the ensemble-mean and the spread from different observational estimates (e.g., 14, 15, and Fig.1 here). The values of regional trends in OHC and thermosteric sea level rise mentioned in the manuscript are only discussed if the discrepancies between observations and GF estimates are larger than the error estimates derived here.

D. Error estimates: observational products. There is a vast literature describing observational products and associated errors (e.g., 16–18). In addition to the sparsity of the data, especially at high latitudes and in the early part of the historical record, there are other factors leading to uncertainty in OHC estimates: error related to the measurements themselves (i.e., instrumental error), and errors due to the methods used to filling the gap in data sampling. The methods include infilling of data gaps via statistical methods, which often relies on knowledge of temporal and spatial covariance of the data. The uncertainty associated with mapping techniques has been well documented in previous studies (18, 19). Other methods to cover the gap in sampling is to rely on data assimilation techniques, which combines observations with a numerical model –none used in the present study (20). As shown in Fig. 1, there are substantial differences among the observationally-based estimates using direct in-filling. Our GFs estimates are often situated within the bounds of the different products, except perhaps for the early part of the record – however error uncertainty estimates might also be underestimated in all products. To easily compare with observations, we have presented the observational linear trends in Fig. 1 (and associated discussion in the main manuscript) as an ensemble-mean, with the error given by the one standard deviation. This type of quantification of uncertainty estimate is likely optimistic, as discussed by (4), especially given that the uncertainty associated with the sparsity of data in the earlier part of the record are not adequately represented by such an unbiased uncertainty quantification.

E. Timeseries of OHC as a function of latitudes. Heat redistribution by changes in ocean circulation integrates to zero globally; a property that is respected by the use of GFs. However, as shown in Fig. 3, a signature of ocean circulation change is present on a regional scale in the North Atlantic. Since the OHC trends are not necessarily linear, and exhibit strong variability on a wide range of timescales (Figs. 1 and 3), let us consider the temporal evolution of OHC. In the Southern Ocean between 80° S and 60° S, there are weak trends over 1955-2017 in both observations (Figs. S3, grey shading representing observational estimates) and GFs (orange curves). Note that the lack of trends in the Southern Ocean could be due to lack of observations (21). Between 60° S and 40° S, the increase in heat storage is weaker in the GF estimates than that observed (0.03 ZJ/°lat), yet still within observational uncertainty. There is a warming trend at all latitudes ranging from 60° S to 20° N in both GF estimates and observations, with magnitudes of 1-2 and 0.5-1 ZJ/°lat, respectively, occurring in the upper 2000 m over the last 60 years. Between 20° N and 50° N, discrepancies in trends and variability between the GF and observational estimates are further discernible, indicating strong changes in ocean transport on all timescales.

At high latitudes in both hemispheres, there is a signature of decadal variability in the upper 2000 m, rather than distinct warming trends. Between 80° S and 60° S in the Southern Ocean, the low-frequency variability in the GF and observational timeseries are anti-correlated (Fig. S3), indicating a role for ocean circulation change on decadal timescales in the Weddell and Ross Sea regions and north of it as the water enters the Atlantic and Pacific Oceans. In observations, this variability is significant in the South Atlantic south of 40° S, with an amplitude of up to 0.3 ZJ/°lat, while in the GF estimates only south of 60° S is the decadal variability (on the order of 0.2 ZJ/°lat) dominating the linear trend. In the North Atlantic, the GF-inferred variability is substantial in both the subtropical and subpolar gyres, and can be comparable to the trend, as discussed in the main text. Decadal variability dominates north of 50° N with no obvious detectable warming or cooling trends (similarly to SSTs, Fig. S1) in observations and GF estimates. However, the magnitude of North Atlantic OHC changes in observations is rather different than in GF estimates. Figs. 3 and S3 are therefore consistent and ocean transport must have been altered to explain the observed patterns of warming north of 20° S. However, the cause ocean transport changes remain to be further analyzed, in particular the contribution of natural variability and anthropogenic forcing.

1. Wunsch C (2011) The decadal mean ocean circulation and sverdrup balance. *Journal of Marine Research* 69(2-3):417–434.
2. Forget G, et al. (2015) Ecco version 4: An integrated framework for non-linear inverse modeling and global ocean state estimation. *Geosc. Model Dev.* 8(10):3071.
3. Thomas MD, De Boer AM, Johnson HL, Stevens DP (2014) Spatial and temporal scales of sverdrup balance. *Journal of Physical Oceanography* 44(10):2644–2660.
4. Wunsch C (2016) Global ocean integrals and means, with trend implications. *Annual Review of Marine Science* 8(1):1–33.
5. Graven HD, Gruber N, Key R, Khatiwala S, Giraud X (2012) Changing controls on oceanic radiocarbon: New insights on shallow-to-deep ocean exchange and anthropogenic CO_2 uptake. *J. Geophys. Res.* 117.
6. Khatiwala S, Graven H, Payne S, Heimbach P (2018) Changes to the air-sea flux and distribution of radiocarbon in the ocean over the 21st century. *Geophysical Research Letters* 45(11):5617–5626.
7. Khatiwala S, et al. (2013) Global ocean storage of anthropogenic carbon. *Biogeosciences.*
8. Wunsch C, Heimbach P (2014) Bidecadal Thermal Changes in the Abyssal Ocean. *J. Phys. Oceanogr.* 44(8):2013–2030.
9. Purkey SG, Johnson GC (2010) Warming of global abyssal and deep southern ocean waters between the 1990s and 2000s: Contributions to global heat and sea level rise budgets. *Journal of Climate* 23(23):6336–6351.
10. Khatiwala S, Primeau F, Holzer M (2012) Ventilation of the deep ocean constrained with tracer observations and implications for radiocarbon estimates of ideal mean age. *Earth and Planetary Science Letters* 325:116–125.
11. Karspeck AR, et al. (2017) Comparison of the atlantic meridional overturning circulation between 1960 and 2007 in six ocean reanalysis products. *Climate Dynamics* 49(3):957–982.
12. Huang B, et al. (2015) Extended reconstructed sea surface temperature version 4 (ersst. v4). part i: upgrades and intercomparisons. *Journal of climate* 28(3):911–930.
13. Hirahara S, Ishii M, Fukuda Y (2014) Centennial-scale sea surface temperature analysis and its uncertainty. *Journal of Climate* 27(1):57–75.
14. Hartmann D, et al. (2013) *Observations: Atmosphere and Surface*, eds. Stocker T, et al. (Cambridge University Press, Cambridge, United Kingdom and New York, NY, USA), pp. 159–254.
15. Church J, et al. (2013) *Sea Level Change*, eds. Stocker T, et al. (Cambridge University Press, Cambridge, United Kingdom and New York, NY, USA), pp. 1137–1216.
16. WCRP Global Sea Level Budget Group (2018) Global sea level budget 1993–present. *Earth Syst. Sci. Data* 10:1551–1590.
17. Abraham JP, et al. (2013) A review of global ocean temperature observations: Implications for ocean heat content estimates and climate change. *Reviews of Geophysics* 51(3):450–483.
18. Boyer T, et al. (2016) Sensitivity of global upper-ocean heat content estimates to mapping methods, xbt bias corrections, and baseline climatologies. *Journal of Climate* 29(13):4817–4842.
19. Cheng L, et al. (2017) Improved estimates of ocean heat content from 1960 to 2015. *Science Advances* 3(3):e1601545.
20. Storto A, et al. (2017) Steric sea level variability (1993–2010) in an ensemble of ocean reanalyses and objective analyses. *Climate Dynamics* 49(3):709–729.
21. Gille ST (2002) Warming of the southern ocean since the 1950s. *Science* 295(5558):1275–1277.

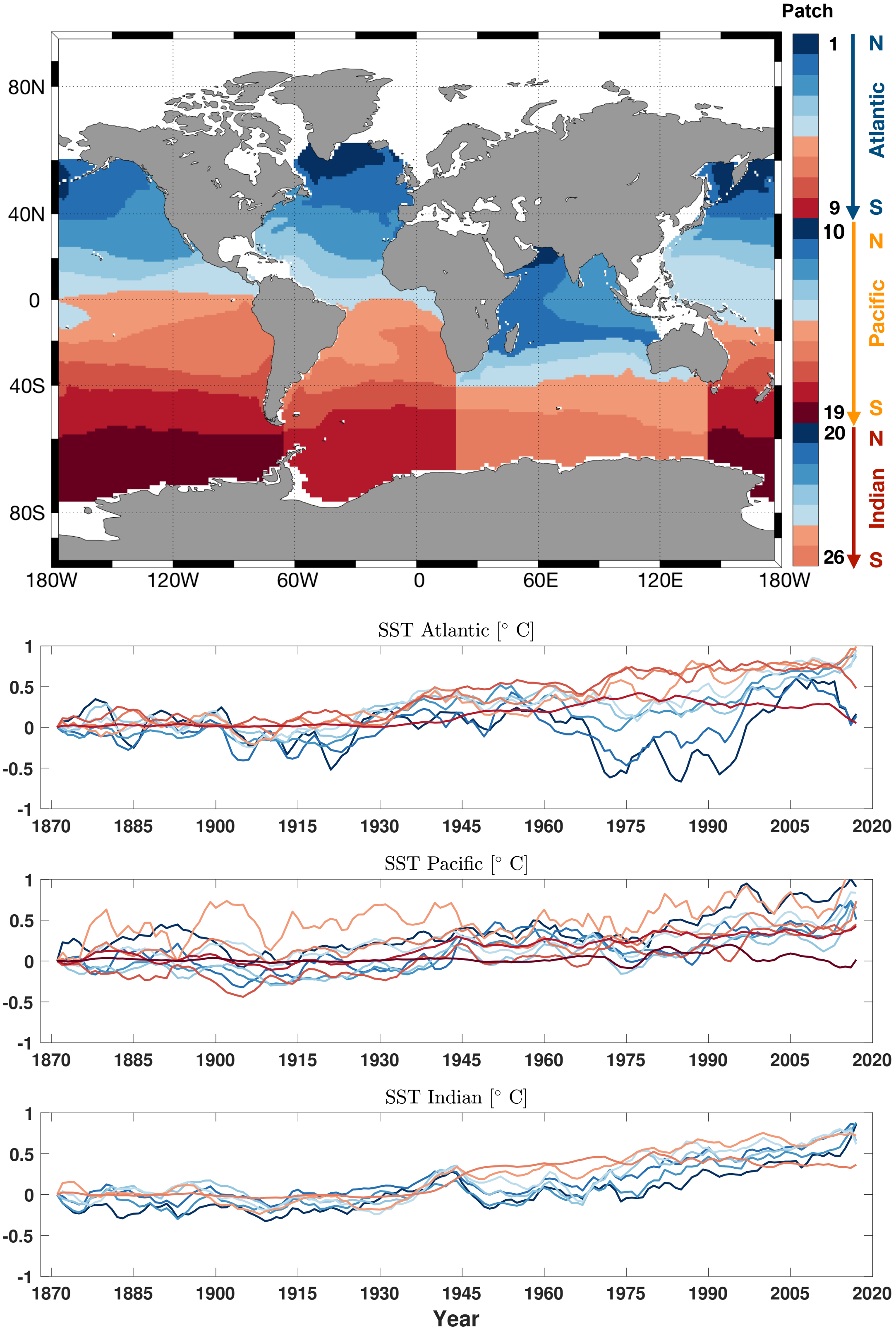

Fig. S1. Areas for the 26 surface patches used for the SST anomalies, and associated area-weighted timeseries from HadISST1 for individual patches in the Atlantic Ocean (top), Pacific Ocean (middle), and Indian Ocean (bottom). The colorbar is associated with the patch number in each basin.

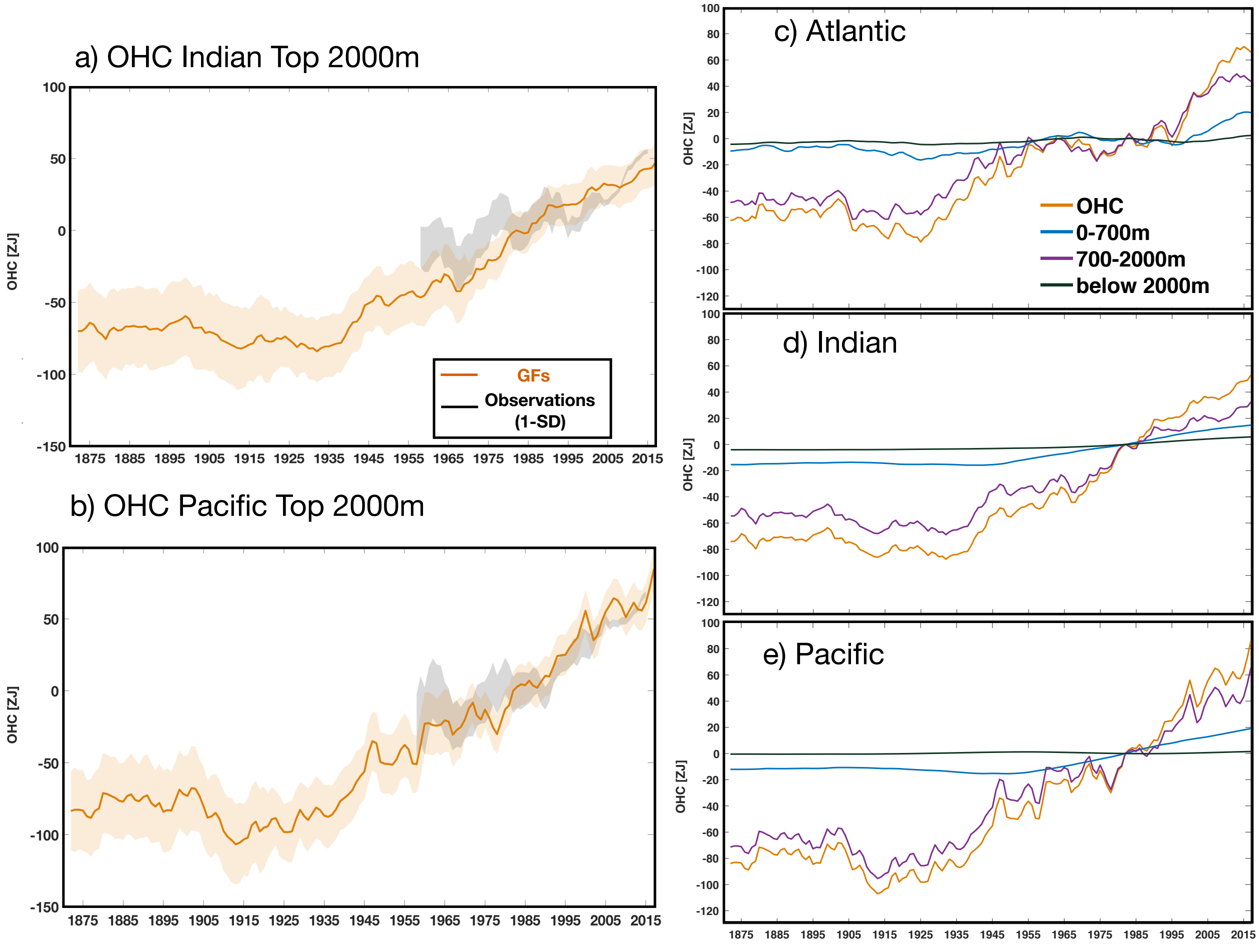

Fig. S2. Timeseries of OHC change in ZJ $(1\text{ZJ} = 10^{21}\text{J})$. Change, relative to 2006-2014, for the top 2000 m in the a) Indian Ocean, and b) Pacific Ocean for the GFs estimates (orange) and observational estimates (grey). GFs estimates, relative to 1971, in the c) Atlantic basin; d) Indian basin; e) Pacific basin for different depth ranges: orange = full depth; purple = 0-700 m ; blue = 700-2000 m; green = below 2000 m.

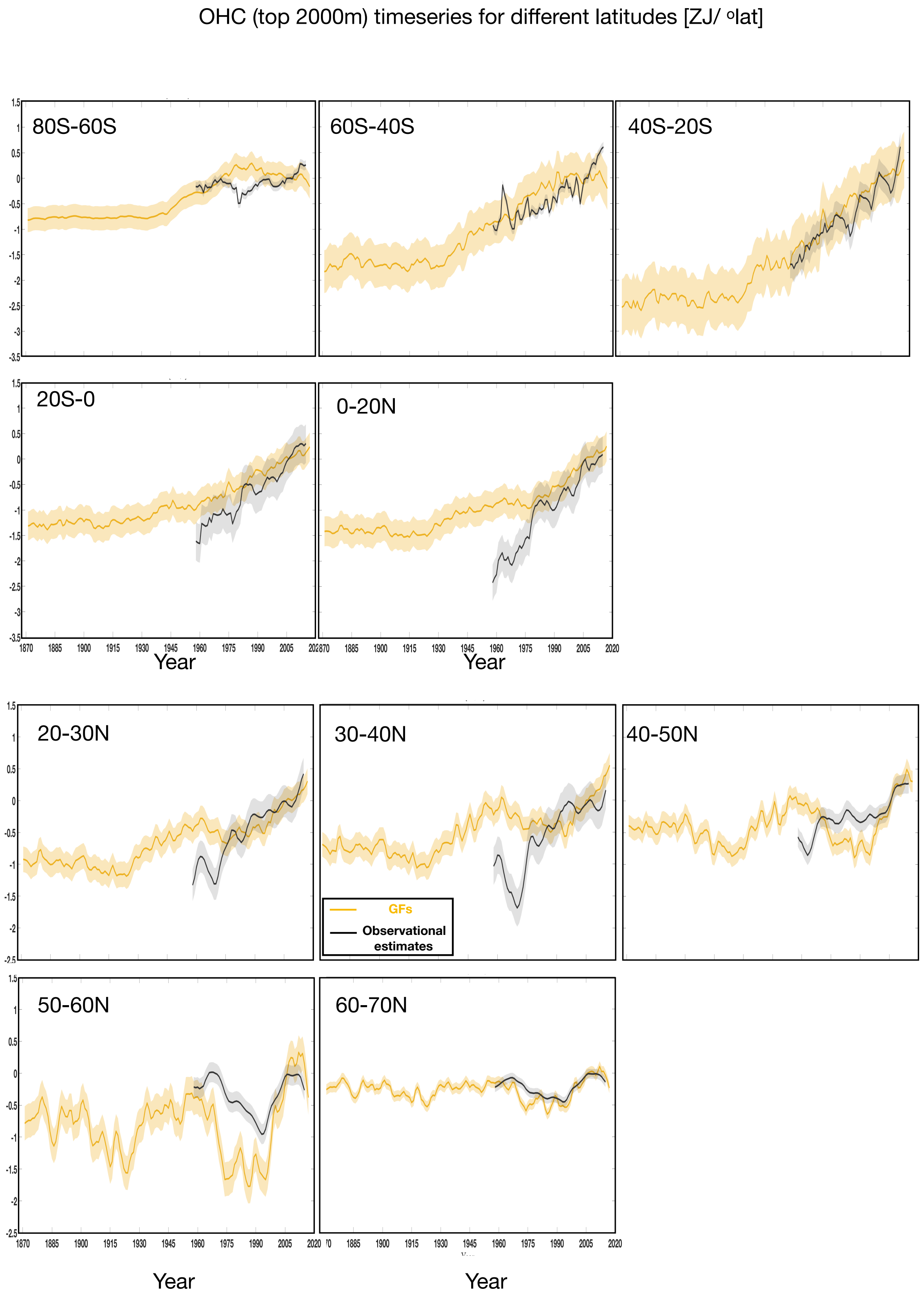

Fig. S3. Timeseries of Atlantic OHC in the upper 2000 m, in $[\mathrm{ZJ}\ /^{\circ}\mathrm{lat}]$, using 20° or 10° latitudinal bands. This includes the reconstruction based on GFs (orange) and the ensemble mean of all observational estimates (black), and associated uncertainty (shading).

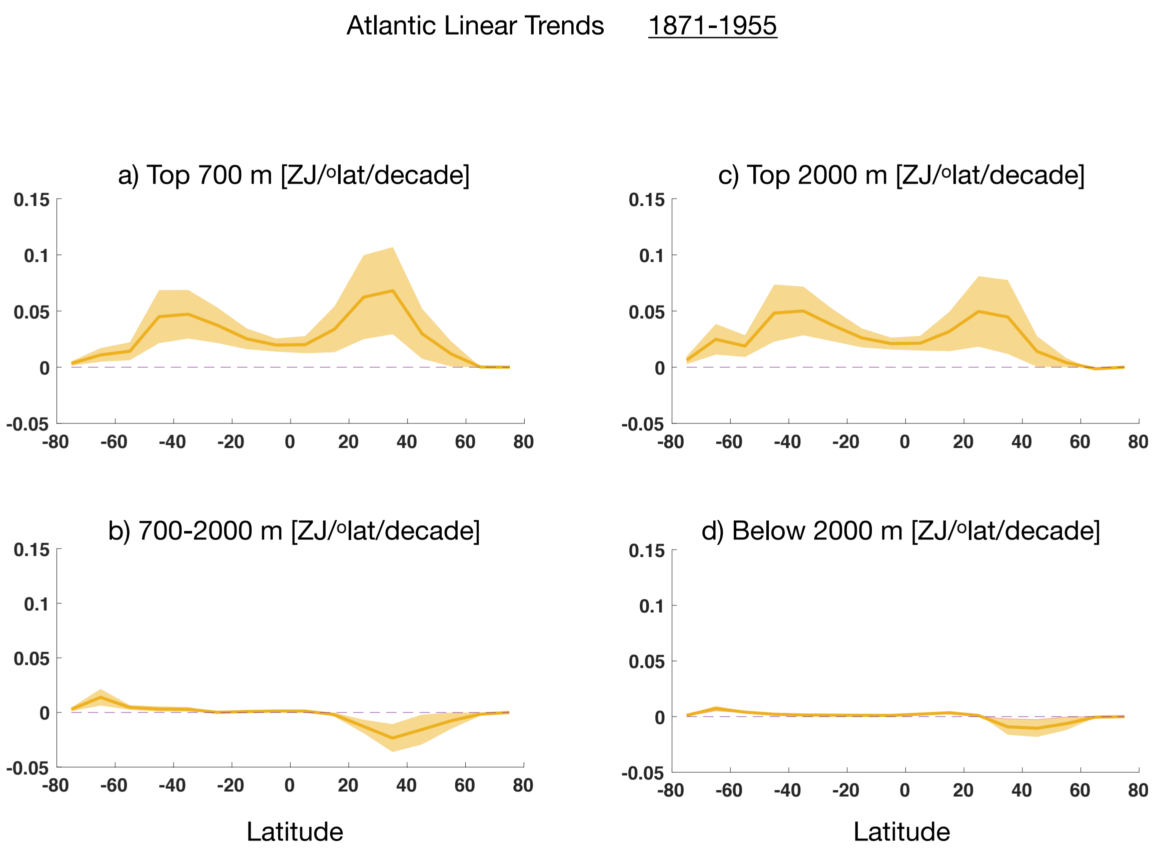

Fig. S4. Atlantic OHC GFs trends for 1871 to 1955 as a function of latitudes, as in Fig 3A-D: a) Top 700 m; b) 700-2000 m; c) top 2000 m; d) below 2000 m.

Zanna et al. 10.1073/pnas.1808838115
